# Hypusination in intestinal epithelial cells protects mice from infectious colitis

**DOI:** 10.1080/19490976.2024.2438828

**Published:** 2024-12-14

**Authors:** Alain P. Gobert, Caroline V. Hawkins, Kamery J. Williams, Lydia A. Snyder, Daniel P. Barry, Mohammad Asim, Margaret M. Allaman, Kara M. McNamara, Alberto G. Delgado, Yu Wang, Shilin Zhao, Kristie L. Rose, M. Blanca Piazuelo, Keith T. Wilson

**Affiliations:** a Division of Gastroenterology, Hepatology, and Nutrition, Department of Medicine, Vanderbilt University Medical Center, Nashville, TN, USA; b Center for Mucosal Inflammation and Cancer, Vanderbilt University Medical Center, Nashville, TN, USA; c Program in Cancer Biology, Vanderbilt University Medical Center, Nashville, TN, USA; d Department of Biostatistics, Vanderbilt University Medical Center, Nashville, TN, USA; e Department of Biochemistry, Mass Spectrometry Research Center, Vanderbilt University School of Medicine, Nashville, TN, USA; f Department of Pathology, Microbiology, and Immunology, Vanderbilt University Medical Center, Nashville, TN, USA; g Veterans Affairs Tennessee Valley Healthcare System, Nashville, TN, USA

**Keywords:** Polyamines, hypusine, translation, attaching and effacing pathogen, Enteropathogenic *Escherichia coli*, Citrobacter rodentium, colitis, mucosal immune response, host–pathogen interactions

## Abstract

Enteropathogenic *Escherichia coli* (EPEC) is a bacterium that causes attaching/effacing (A/E) lesions and serious diarrheal disease, a major health issue in developing countries. EPEC pathogenicity results from the effect of virulence factors and dysregulation of host responses. Polyamines, including spermidine, play a major role in intestinal homeostasis. Spermidine is the substrate for deoxyhypusine synthase (DHPS), which catalyzes the conjugation of the amino acid hypusine to eukaryotic translation initiation factor 5A (EIF5A); hypusinated EIF5A (EIF5A^Hyp^) binds specific mRNAs and initiates translation. Our aim was to determine the role of hypusination during infection with A/E pathogens. We found that DHPS and EIF5A^Hyp^ levels are induced in i) a colonic epithelial cell line and human-derived colon organoids infected with EPEC, and ii) the colon of mice infected with *Citrobacter rodentium*, the rodent equivalent of EPEC. Specific deletion of *Dhps* in intestinal epithelial cells worsened clinical, histological, and pro-inflammatory parameters in *C. rodentium*-infected mice. These animals also exhibited an exacerbated pathogenic transcriptome in their colon. Furthermore, infected mice with specific *Dhps* deletion exhibited reduced levels of proteins involved in detoxification of tissue-damaging reactive aldehydes and consequently increased electrophile adducts in the colon. Thus, hypusination in intestinal epithelial cells protects from infectious colitis mediated by A/E pathogens.

## Introduction

*Escherichia coli* is a major commensal from the resident intestinal microbiota, but also one of the most prevalent food contaminant microorganisms worldwide. Pathogenic *E. coli* have been classified into six diarrheagenic pathobionts based on their effects on human health.^1^ Among them, enteropathogenic *E. coli* (EPEC) is a major cause of infant diarrhea in developing countries.^2^ Despite the development of proper therapeutic interventions and improving sanitary conditions, EPEC remains the first bacterial cause of death by diarrhea in the world.^3^

The molecular mechanisms by which EPEC colonizes the human gastrointestinal tract and causes diarrhea have been well established.^4^ First, the type IV bundle forming pilus allows EPEC to attach to intestinal epithelial cells (IECs^5^). Then, the bacteria directly inject an effector protein called translocated intimin receptor (Tir) into the host cell through a type 3 secretion system, which is encoded by genes belonging to the locus of enterocyte effacement (LEE). Tir inserts into the cell membrane so that the *N*- and C-terminal portions remain cytosolic, whereas the central domain traverses the host membrane, serving as a receptor for the EPEC intimin, encoded by the *eae* gene. Tir is also phosphorylated on tyrosine 474,^6^ leading to the recruitment of the adaptor NCK and formation of the NCK/N-WASP/ARP2/3/actin complex.^7^ Consequently, a network of actin filaments forms underneath the bacteria, leading to pedestal formation and effacement of brush border microvilli; this unique histopathological lesion is known as an attaching and effacing (A/E) lesion. Then, loss of microvilli, disruption of tight junction integrity, tissue damage due to prolonged mucosal inflammation, and dysregulation of ion exchange support the pathogenicity of the infection.^8,9^ Although the molecular aspects of EPEC virulence have been well defined, a better understanding of the host determinants that can regulate the infection and the pathogenicity may help for the development of novel therapeutic approaches. Interestingly, the murine pathogen *Citrobacter rodentium* uses the same molecular tools as EPEC to colonize the intestine, initiate A/E lesions, and induce colon inflammation, therefore representing a tool of choice to study the crosstalk between A/E pathogens and the host.^10^

Our group has reported that the dysregulation of polyamine metabolism in the colon affects *C. rodentium* pathogenicity.^11–13^ The three biogenic polyamines, putrescine, spermidine (Spd), and spermine play key functions in a variety of cellular processes, from maintenance of DNA structure to post-translational regulation, and are thus essential for cellular homeostasis.^14^ Putrescine is synthesized by ornithine decarboxylase (ODC1, also known as ODC) from ornithine and is then sequentially converted into Spd and spermine via the action of Spd synthase and spermine synthase, respectively. Spd is essential to support the translation of specific proteins through a mechanism termed hypusination. The enzyme deoxyhypusine synthase (DHPS) transfers the N-terminal moiety of Spd to the lysine-50 residue of the protein eukaryotic translation initiation factor 5A (EIF5A) to form the intermediate deoxyhypusine residue, which is then hydroxylated by deoxyhypusine hydroxylase (DOHH) to complete the synthesis of the nonproteinogenic amino acid hypusine.^15,16^ Hypusinated EIF5A (EIF5A^Hyp^) enters the nucleus and binds specific mRNAs, notably those exhibiting a 5′-AAAUGU-3′ consensus sequence.^17,18^ EIF5A^Hyp^/mRNA complexes are then translocated to the cytoplasm by the nuclear transporter exportin-4^19^ and reach the ribosomes for translation.^20^ EIF5A^Hyp^ also alleviates ribosome pausing during the translation of peptides enriched in diprolyl and diglycyl motifs.^21,22^ Hypusination is essential for embryogenesis and deletion of *Eif5a1* or *Dhps* is lethal in mice.^23^

We previously reported that hypusination in myeloid cells is required to limit the colonization of pathogenic bacteria in the gastrointestinal tract, including *Helicobacter pylori* in the stomach and *C. rodentium* in the colon.^24^ The aim of the present study was to investigate the role of epithelial hypusination on pathogenicity of A/E pathogens. We found herein that hypusination is increased in human colonic epithelial cells (CECs) stimulated with EPEC and in the colon of *C. rodentium*-infected mice. Moreover, mice with specific deletion of *Dhps* in IECs were more colonized by *C. rodentium* and developed more severe colitis. We further evidenced that hypusination regulates the translation of proteins implicated in the detoxification of deleterious reactive aldehydes in the infected mucosa.

## Materials and methods

### Ethical statement

The study protocol regarding collection of human colon biopsy samples during colonoscopy at Vanderbilt University Medical Center was approved by the Vanderbilt University Institutional Review Board (IRB protocol 211,235). Written informed consent was obtained from all participants.

Experiments with mice were conducted under protocol M20000047 that was approved by the Vanderbilt University IACUC and Institutional Biosafety Committee, and the Research and Development Committee of the Veterans Affairs Tennessee Valley Healthcare System. Procedures were performed in accordance with institutional policies, AAALAC guidelines, the AVMA Guidelines on Euthanasia, NIH regulations (Guide for the Care and Use of Laboratory Animals), and the United States Animal Welfare Act (1966).

### Bacteria

We used *C. rodentium* strain DBS100,^25^ a human-derived *eae*^+^ EPEC strain (ATCC 43,887), and the EPEC strain E2348/69 with its isogenic mutants for *escN*, *tir*, and *nleA*. These bacteria were maintained on Luria Bertani (LB) agar plates. Isolated colonies grown in LB broth until the exponential phase were used for infection of mice and cells.

### Human cells

The colorectal cancer cell line HCT 116 (ATCC CCL-247) was maintained in DMEM containing 10% fetal bovine serum, 10 mm HEPES, 1 mm sodium pyruvate and penicillin–streptomycin mix. For the experiments, 5 × 10^5^ cells were plated in 24-well plates without antibiotics for 24 h and then infected for 6 h with EPEC at a multiplicity of infection of 1.

The generation of colon organoids was performed as reported.^26^ The colonoid line DoD022 was isolated from normal tissue from a sigmoid colon biopsy from a 47-year-old female patient undergoing colonoscopy. It was maintained in Matrigel Matrix (Corning) overlaid with IntestiCult Organoid Growth Medium (Stemcell Technologies) containing penicillin-streptomycin and the GSK3 inhibitor CHIR 99,021 (4 mm; Tocris). For the experiments, cells were removed from Matrigel using cold PBS, resuspended in Cell Recovery Solution (Corning), washed, diluted in Intesticult Organoid Growth Media plus 4 μM CHIR 99,021, and added (2 × 10^5^ per well) to 24-well plates pre-coated with Matrigel (1:100 in PBS). After 24 h, colonoids were pre-treated with the DHPS inhibitor N1-guanyl-1,7-diaminoheptane (GC7) for 2 h and then infected with EPEC at an MOI of 1 for 6 h.

To determine adhesion and internalization of EPEC, human CECs were washed thoroughly with PBS, incubated or not for 1 h with 200 μg/ml gentamicin, counted, and lysed in 0.1% saponin for 30 min at 37 °C. The number of bacteria in each lysate was determined by counting the CFUs after plating serial dilutions on LB plates.

### Animals and infection

Littermate co-housed *Dhps*^*fl/fl*^ and *Dhps*^*fl/fl*^;*Vil1*^*cre/+*^ mice (*Dhps^Δ^*^*epi*^) female and male mice^26^ were house-bred in our animal facility. Mice were infected with 5 × 10^8^ *C. rodentium* DBS100^12,13^ in 100 μl LB broth. Mice were weighed and monitored daily and those that showed extreme distress or turned out to be moribund were sacrificed. Experiments were ended at day 14 post-infection. Colons were removed, measured, cut longitudinally, cleaned, weighed, and Swiss-rolled for histology. Three proximal and distal 2 mm pieces were used for RNA and protein analysis and to determine colonization by culturing serial dilution of ground tissues on MacConkey agar plates, on which *C. rodentium* form small, circular, and pink colonies. Colonization was also assessed in homogenates of the spleen.

### Histologic injury scores

Sections of colons fixed in formalin and embedded in paraffin were stained with hematoxylin and eosin (H&E) and examined in a blinded manner by gastrointestinal pathologists (M.B.P). For *C. rodentium* colitis, the histologic injury score (0–36) was the sum of *i*) acute and chronic inflammation scores (0–3) multiplied by extent of inflammation (0–4) plus *ii*) the epithelial injury score (0–3) multiplied by extent of epithelial injury (0–4).

### Isolation of CECs

CECs were isolated by a dissociation and dispersion method. Briefly, the colon was opened longitudinally, cleaned, cut into 5 mm pieces, and incubated in 25 ml of PBS containing 3 mm DTT, and 0.5 mm EDTA. After one hour at 4°C, tissues were transferred into 10 ml PBS, vigorously shaken, and filtered through 70-μm nylon mesh; this was repeated 3 times, and the fractions were combined. Samples were centrifuged and the cells were resuspended in PBS and used for the proteomic analysis.

### Analysis of mRNA expression

Total RNA from colonic tissues was isolated using the RNeasy Mini kit (Qiagen). Quality control of each sample, library preparation of cDNA, and Next Generation Sequencing (PE150) was performed using TapeStation (Agilent), the NEBNext^Ⓡ^ Ultra™ II Directional RNA Library Prep Kit for Illumina^Ⓡ^ (BioLabs, Inc.), and Illumina NovaSeq6000 with NovaSeq 6000 SP Reagent Kit (Illumina), respectively. Reads were trimmed to remove adapter sequences using Cutadapt v4.5) and aligned to the Gencode GRCm38.p6 genome using STAR (v2.7.11a).^27^ Gencode vM24 gene annotations were provided to STAR to improve the accuracy of mapping. Quality control on both raw reads and adaptor-trimmed reads was performed using FastQC (v0.12.1; www.bioinformatics.babraham.ac.uk/projects/fastqc). FeatureCounts (v2.0.6) was used to count the number of mapped reads to each gene.^28^ Heatmap3 was used for cluster analysis and visualization.^29^ Significantly differentially expressed genes (DEGs) with absolute fold change > 1.5 and FDR adjusted *p* value ≤ 0.05 were detected by DESeq2 (v1.40.2).^30^ Genome Ontology and KEGG pathway over-representation analysis was performed on DEGs using the WebGestaltR package (v0.4.6). Gene set enrichment analysis was performed using GSEA package (v4.3.2) on database (v2022.1.Hs).

Reverse transcription was performed using Superscript II Reverse Transcriptase and Oligo dT; then mRNAs were amplified using the PowerUp SYBR Green Master Mix and the following primers: Murine *Actb*: F, CCAGAGCAAGAGAGGTATCC and R, CTGTGGTGGTGAAGCTGTAG; murine *Cxcl1*: F, GCTGGGATTCACCTCAAGAA and R, CTTGGGGACACCTTTTAGCA; murine *Nos2*: F, CACCTTGGAGTTCACCCAGT and R, ACCACTCGTACTTGGGATGC; murine *Tnf*: F, CTGTGAAGGGAATGGGTGTT and R, GGTCACTGTCCCAGCATCTT; murine *Il17*: F, ATCCCTCAAAGCTCAGCGTGTC and R, GGGTCTTCATTGCGGTGGAGAG. *Actb* gene was used as housekeeping gene and the semi-quantitative analysis was performed using the mean of all the untreated *Dhps*^*fl/fl*^ mice as the reference sample.

### Protein analysis

Quantitative proteomics was performed as described.^31^ Briefly, protein extracts (30 μg) containing 5% SDS were reduced with 10 mm tris(2-carboxyethyl) phosphine, alkylated with 20 mm iodoacetamide, treated with 1.2% aqueous phosphoric acid, washed by 6 volumes of 90% methanol containing 100 mm tetraethylammonium bromide (TEAB), and loaded on S-Trap micro spin column (ProtiFi). After 4 washes with 90% methanol containing 100 mm TEAB, samples were digested with trypsin gold (Promega) at a 1:10 ratio in 50 mm TEAB. Peptides were eluted using 50 mm TEAB, 0.2% formic acid, and then 0.2% formic acid in 50% acetonitrile, concentrated by speed-vac, and dissolved in 0.2% formic acid. Samples were analyzed by LC-coupled tandem mass spectrometry on an Orbitrap Exploris 480 mass spectrometer (Thermo Scientific). For identification of peptides, data were searched with Maxquant, version 2.0.3.0.^32^ MS/MS spectra were searched with the Andromeda search engine^33^ against a mouse database created from the UniprotKB protein database with default Maxquant protein contaminants added. Default parameters were used for Maxquant, with the addition of LFQ and match between runs. Variable modifications included methionine oxidation and N-terminal acetylation, and carbamidomethyl cysteine was selected as a fixed modification. The false discovery rate (FDR) was set to 0.01 for peptide and protein identifications. Label-free quantitative analysis of identified proteins was performed with the MSstats R package,^34^ version 4.8.7, using default parameters which include the following: equalize medians for the normalization method, Log2 transformation, Tukey’s median polish as the summary method, and model-based imputation.

The Western blots were performed as described.^24,26^ We used a rabbit polyclonal anti-DHPS antibody (Ab) (Abcam Cat # AB202133; 1:2000), a rabbit polyclonal anti‑EIF5A^Hyp^ Ab (Millipore, Cat # ABS1064; 1:5000), a mouse monoclonal anti‑EIF5A Ab (BD Transduction Laboratories, Cat # 611977; 1:6,000), a rabbit polyclonal anti‑GSTO1 Ab (LifeSpan BioSciences, Cat # C314575; 1:1,000), a rabbit polyclonal anti‑GSTP1 Ab (Invitrogen, Cat # PA5–29558; 1:3,000), or mouse monoclonal anti-ACTB Ab (Millipore, Cat # A1978; 1:20,000) as primary antibodies. The Peroxidase AffiniPure Goat Anti-Rabbit IgG (Jackson ImmunoResearch Laboratories Inc., Cat # 111-035-003; 1:5000) and the Peroxidase AffiniPure Goat Anti-Mouse IgG (Jackson ImmunoResearch Laboratories Inc., Cat # 115-035-003; 1:10000) were used as secondary antibodies. Densitometry was performed using ImageJ 1.53a.

### Functional pathway analysis

Ingenuity Pathway Analysis (IPA) software (QIAGEN) was used for the functional interpretation of data obtained from the RNA-Seq and proteomics.

### Immunostaining of the tissues

For immunohistochemistry (IHC), sections were deparaffinized and incubated at room temperature with 3% hydrogen peroxide in phosphate-buffered saline to block endogenous peroxidase and blocked for 1 h in Background Sniper. Slides were then incubated overnight at 4°C with a rabbit monoclonal anti-Ki-67 Ab (Biocare Medical; 1:100) followed by treatment with EnVision+/HRP Labeled Polymer Anti-Rabbit (Agilent Dako) for 1 h at room temperature.^26^ For the immunodetection of adducts of iso-levuglandins (isoLG) to lysine, termed isoLG-lysyl adducts,^26^ tissue sections were sequentially incubated with 3% hydrogen peroxide in PBS, a solution of 5% human/mouse serum and 5% BSA in PBS, the D11 Ab (1:250), the DYKDDDDK Epitope Tag Ab (1:500; Novus), and the EnVision+/HRP Labeled Polymer Anti-Rabbit (Agilent Dako). Detection was performed using 3,3′-diaminobenzidine, and tissues were counterstained by hematoxylin.

The expression of the tight junction protein zonula occludens-1 (ZO-1) was assessed by immunofluorescence.^26^ Deparaffinized sections of the colon tissues were sequentially treated with the Rodent Block M (Biocare Medical), an anti-ZO-1 mouse monoclonal Ab (Invitrogen; 1:200) overnight at 4°C, and with a goat anti-Mouse IgG (H+L) Highly Cross-Adsorbed Secondary Antibody, Alexa Fluor™ Plus 488 (Invitrogen; 1:600) at 37°C for 45 min; sections were then blocked with Background Sniper (Biocare Medical), incubated overnight at 4°C with anti-human/mouse E-cadherin goat polyclonal Ab (R&D System; 1:100), and then with a donkey anti-Mouse IgG (H+L) Highly Cross-Adsorbed Secondary Antibody, Alexa Fluor™ 555 (Invitrogen; 1:600) at 37°C for 45 min. Slides were mounted with VECTASHIELD HardSet™ Antifade Mounting Medium with DAPI (Vector Laboratories) and visualized using a Nikon E800 microscope and a SPOT Imaging CMOS camera.

### Measurement of cytokines and chemokines

Colonic tissues were lysed in Cell Lytic Mammalian Tissue Lysis Extraction Reagent (Sigma) and protein concentrations were measured by the BCA Protein Assay Kit (Pierce). The analysis was performed using the MILLIPLEX Mouse Cytokine/Chemokine Magnetic Bead Panel (Millipore; Cat # MCYTOMAG-70K-PX32) on a FlexMap 3D Instrument. Data were standardized to tissue protein concentrations.

### Statistics

Figures and statistics were performed using GraphPad Prism 10.4.0 software. Significance level was set as *p* < 0.05 and all statistical tests were two-sided. All the data represent the mean ± SEM. Outliers were identified using the ROUT test (Q = 5%) and removed from the analysis. Data that were not normally distributed according to the D’Agostino & Pearson normality test were log or square root transformed, and distribution was re-assessed. Student’s *t* test was used to determine significant differences between two groups. Multiple comparisons were performed using one-way ANOVA with the Tukey, Dunnett, or Holm-Šídák’s test.

## Results

### *C. rodentium* infection stimulates hypusination in epithelial cells

**Figure 1.**
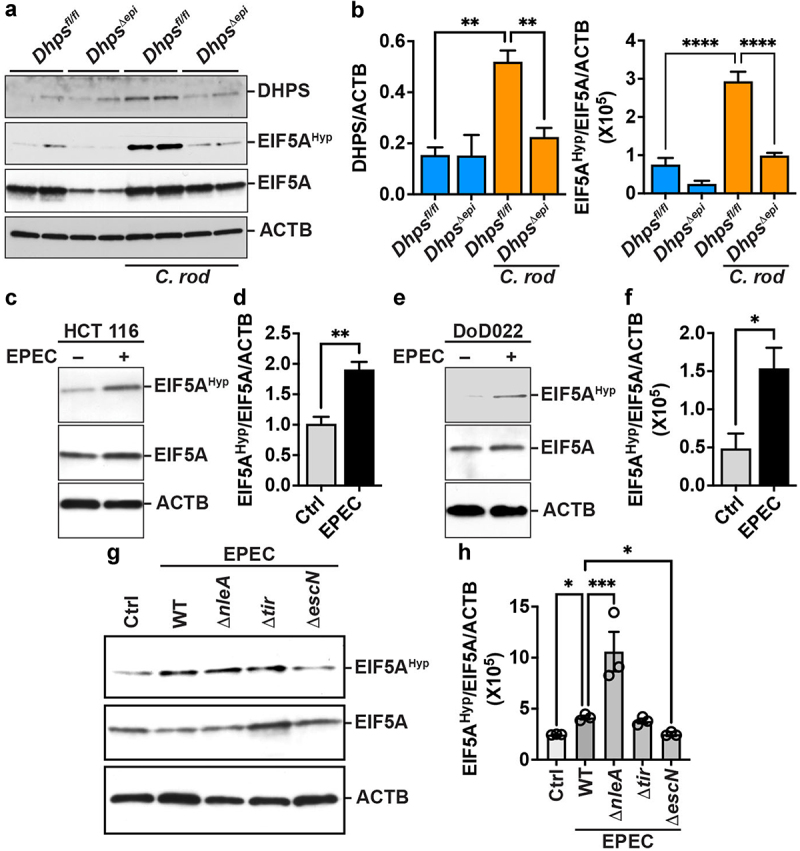
Induction of the DHPS/EIF5A^Hyp^ pathway by A/E pathogens. (a,b) *Dhps*^*fl/fl*^ and *Dhps*^*∆epi*^ mice were infected or not with *C. rodentium* for 14 days. Colons (*n* = 4 mice per group) were removed and proteins were extracted from the whole tissue. DHPS and EIF5A^Hyp^ levels were assessed by Western blot (a) followed by densitometric analyses (b). (c,d) the human colon cell line HCT 116 was infected for 6 h with EPEC, and the level of hypusination and EIF5A was determined by Western blotting (c) and densitometry (d). (e,f) the level of EIF5A^Hyp^ and EIF5A in the normal human colonoid line DoD022, infected or not with EPEC for 6 h, was assessed by Western blotting (e) and densitometry (f). The level of EIF5A^Hyp^ and EIF5A was assessed by Western blotting (g) followed by densitometry (h) in the normal human colonoid line DoD022 infected or not with EPEC E2348/69, Δ*nleA*, Δ*tir*, or Δ*escN* for 6 h. Representative data of 3 independent experiments. *p < 0.05, **p < 0.01, ***p < 0.001 by one-way ANOVA and Tukey test (b, g, h) or by unpaired *t* test; C, E, and G are representative data of 3 independent experiments.

We previously reported that infection with *C. rodentium*, the rodent equivalent of EPEC, which causes colitis in mice,^11,35^ leads to the induction of DHPS in colonic macrophages.^24^ Herein, we observed that the protein DHPS and the level of hypusinated EIF5A were both increased in the colon of *Dhps*^*fl/fl*^ mice infected with *C. rodentium* compared to control animals (Figure 1a). As expected, the level of DHPS expression and EIF5A^Hyp^ was markedly reduced in mice with specific deletion of *Dhps* in IECs; there was also a modest reduction of EIF5A level in the colon of uninfected *Dhps^Δ^*^*epi*^ mice. The densitometric analysis indicated that the level DHPS and EIF5A^Hyp^ were significantly increased in *C. rodentium*-infected *Dhps*^*fl/fl*^ mice compared to uninfected animals and significantly reduced in infected *Dhps^Δ^*^*epi*^ mice (Figure 1b). Moreover, the level of hypusinated EIF5A was also increased in the human colon cancer cell line HCT 116 (Figure 1c,d) and in a human organoid line derived from histologically normal colon tissue (Figure 1e,f) infected by EPEC; EIF5A level was not affected by EPEC infection in these cells (Figure 1c,e). Further, we observed that hypusination was less induced in human cells infected with the *escN* mutant, which lacks a functional T3SS,^36^ compared to the wild-type strain (Figure 1g,h) or to the mutant for *tir* that cannot form A/E lesions^6^; a mutant of *nleA*, which encodes for a non-LEE encoded effector that perturbates intestinal tight junctions through the disruption of cellular protein trafficking,^37^ stimulated a higher level of hypusination than the parental strain (Figure 1g,h).

These data demonstrate that A/E pathogens are potent inducers of hypusination in CECs, through a mechanism requiring the T3SS, but independently of A/E lesion formation.

#### Exacerbation of *C. rodentium*-induced colitis by *Dhps* deletion

**Figure 2.**
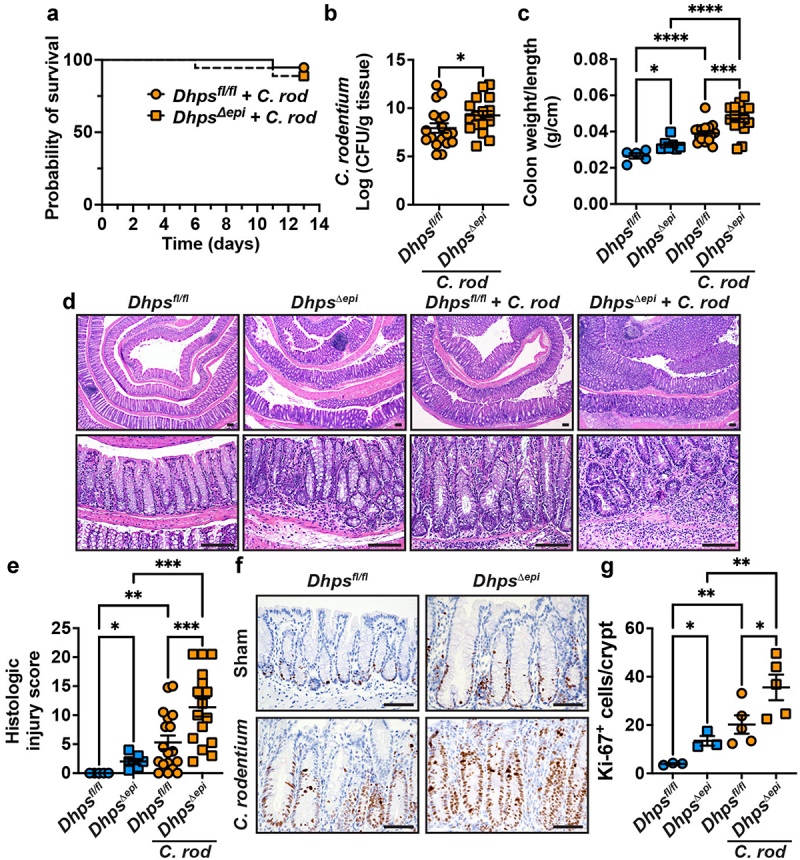
*C. rodentium* colitis in *Dhps*^*fl/fl*^ and *Dhps*^*∆epi*^ mice. Animals were infected or not with *C. rodentium* (*C. rod*) and survival was monitored daily (a); there was no death in the uninfected groups. *P* was calculated by the Log-rank (mantel-cox) test; *n* = 5 *Dhps*^*fl/fl*^ and 7 *Dhps*^*∆epi*^ mice uninfected mice, and *n* = 19 *Dhps*^*fl/fl*^ and 18 *Dhps*^*∆epi*^ infected mice. Animals were sacrificed after 14 days, and colonization was determined by plating serial dilutions of ground colon biopsies (b). The colon weight/length ratio was assessed (c). Colons were Swiss-rolled, stained with H&E (d), and scored for histologic injury (e); scale bar, 50 μm. Proliferation was assessed by IHC for ki-67 ((f); scale bar represents 50 μm) and quantification of ki-67-positive nuclei in CECs (g); positive epithelial cells were counted in 100 crypts along the Swiss roll from *n* = 3 *Dhps*^*fl/fl*^; *n* = 3 *Dhps*^*∆epi*^ mice; *n* = 5 *Dhps*^*fl/fl*^ + *C. rodentium*; *n* = 5 *Dhps*^*fl/fl*^ + *C. rodentium*. in panels with dot plots, *p < 0.05, **p < 0.01, ***p < 0.001, ****p < 0.0001 by one-way ANOVA and Newman–Keuls test (b, c, e) or by one-way ANOVA and Tukey test (G); each dot represents a mouse.

To determine the role of epithelial hypusination in an infectious model of the gastrointestinal tract, we then analyzed the outcome of *C. rodentium* infection in *Dhps*^*fl/fl*^ and *Dhps^Δ^*^*epi*^ mice. Survival was not significantly affected by *Dhps* deletion in IECs during *C. rodentium* infection (Figure 2a). First, colonization of the colon by *C. rodentium* was significantly higher by 1.3-log order in *Dhps^Δ^*^*epi*^ mice compared to *Dhps*^*fl/fl*^ animals (Figure 2b). Colon weight-to-length ratio, a classical marker of colon inflammation, was also significantly increased in *Dhps^Δ^*^*epi*^ versus *Dhps*^*fl/fl*^ mice (Figure 2c), as we reported.^26^ This ratio was also enhanced in infected *Dhps*^*fl/fl*^ mice compared to uninfected animals and was further augmented in *Dhps^Δ^*^*epi*^ mice + *C. rodentium* (Figure 2c). H&E staining of the colon of *C. rodentium*-infected *Dhps*^*fl/fl*^ mice demonstrated an effacement of the brush border, hyperplasia, mucosal inflammation, and submucosal edema (Figure 2d). These features were markedly accentuated in mice with *Dhps* deletion (Figure 2d). Using a comprehensive scoring system to quantify the degree of inflammation and epithelial damage, we confirmed that histologic injury was significantly increased in infected mice from both genotypes versus control animals and that there was more colon injury in *Dhps^Δ^*^*epi*^ mice compared to *Dhps*^*fl/fl*^ animals upon infection (Figure 2e). Note that colon weight-to-length ratio and histologic injury scores were increased in *C. rodentium*-infected *Dhps^Δ^*^*epi*^ mice in both females and males (Figure S1). The expression of the proliferation marker protein Ki-67, assessed by IHC (Figure 2f) and quantification (Figure 2g), was significantly enhanced in naïve *Dhps^Δ^*^*epi*^ mice compared to floxed animals, as previously shown^26^; matching the difference in histology, there was more Ki-67^+^ CECs in *C. rodentium*-infected *Dhps*^*fl/fl*^ and a further and significant increase in mice with specific *Dhps* deletion (Figures 2f,g). Further, in naïve *Dhps*^*fl/fl*^ and *Dhps^Δ^*^*epi*^ mice ZO-1 was localized along the junction of CECs (Figure S2A), as we previously observed.^26^ Infection with *C. rodentium* resulted in a marked redistribution of ZO-1 protein to the apical side of CECs in both genotypes (Figure S2A). In addition, we determined that the number of animals with live *C. rodentium* in the spleen (Figure S2B) and the number of live bacteria in the spleen of animals harboring splenic colonization was similar between *Dhps*^*fl/fl*^ and *Dhps^Δ^*^*epi*^ mice (Figure S2C). These data suggest that *Dhps* deletion in IECs does not favor *C. rodentium* colitis by causing leakiness of the epithelial barrier.

**Figure 3.**
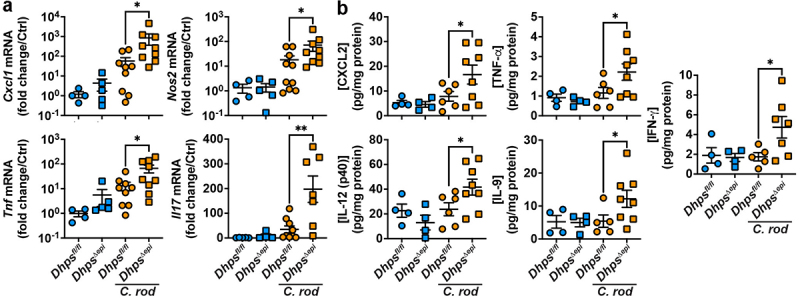
Inflammation in colonic tissues. The expression of the genes *Cxcl1*, *Nos2*, *Tnf*, and *Il17* analyzed by rt-real-time PCR (a) and the measurement of cytokine concentration assessed by luminex (b) were determined in the colon of *Dhps*^*fl/fl*^ and *Dhps*^*∆epi*^ mice, infected or not with *C. rodentium*. *p < 0.05, **p < 0.01 by Dunnett’s multiple comparisons test (a) and Holm-šídák’s multiple comparisons test (b).

The expression of pro-inflammatory genes was analyzed. Specifically, the genes that encode for the chemokine CXCL1, the innate effectors TNF-α and NOS2, and the prototype Th17 cytokine IL-17 were increased in the colon mucosa of infected *Dhps^Δ^*^*epi*^ mice compared to *Dhps*^*fl/fl*^ animals (Figure 3a). Using a Luminex multiplex assay, we found that the chemokine CXCL2 and the pro-inflammatory cytokines TNF-α, IL-12 (p40), IL-9, and IFN-γ were significantly more produced in the colon mucosa of infected *Dhps^Δ^*^*epi*^ mice than in those of infected floxed animals (Figure 3b).

Altogether these data indicate that *Dhps* deletion in IECs worsens *C. rodentium* pathogenicity, suggesting that epithelial hypusination protects from infectious colitis.

#### Development of an exacerbated pathogenic transcriptome in infected *Dhps^Δepi^* mice

We then assessed by RNA-Seq the transcriptomic changes occurring in the colonic mucosa during *C. rodentium* infection and/or *Dhps* deletion. Overall, 33,645 sequences were identified, comprising 18,434 protein coding RNAs. For the analysis of the DEGs between groups, we focused on the genes upregulated or downregulated by 1.5-fold or more and with a *p* < 0.05. The DEGs are listed in Table S1.

**Figure 4.**
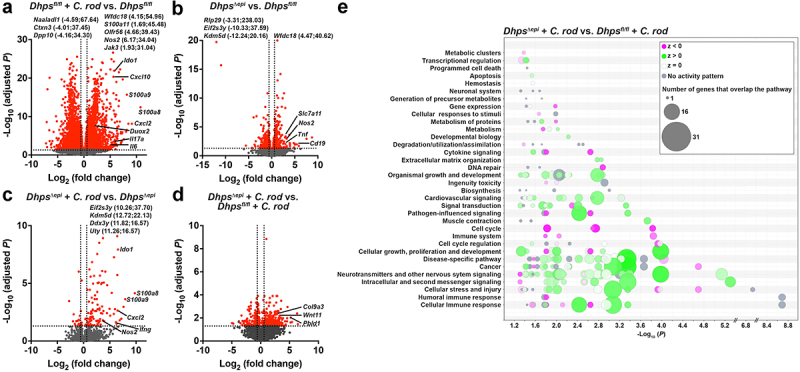
Transcriptomic changes orchestrated by DHPS in the infected mucosa. RNA extracted from the colon from *Dhps*^*fl/fl*^ and *Dhps^Δ^*^*epi*^ (*n* = 3 uninfected and 5 infected mice per genotype) at 14 days post-inoculation with *C. rodentium*, was sequenced, and the volcano plots corresponding to the different comparisons were generated (a-d); red dots correspond to genes significantly (*p* < 0.05) upregulated or downregulated by 1.5-fold or more. Genes with values outside the X and/or Y axis limits are given with their (x;y) coordinates. All the DEGs are provided in Table S1. The DEGs identified in (d) were used for the identification of the “canonical” pathways affected by *dhps* deletion in infected mice using IPA (e); the complete list of pathways is shown in Table S2.

In *Dhps*^*fl/fl*^ mice infected with *C. rodentium*, we found 2675 and 2888 genes upregulated and downregulated compared to uninfected animals, respectively (Figure 4a). The most upregulated genes in the colon of infected mice belong to the pro-inflammatory response and include *i*) the calprotectins *S100a8/a9/a11*; *ii*) the innate effectors *Il6*, *Nos2*, *Duox2*, and *Ido1*; *iii*) the chemokines *Cxcl1/2/3/10*; and *iv*) the prototype Th1 cytokine *Ifng* and Th17 cytokine *Il17a* (Figure 4a). Genes that are markers of inflammation, such as *Cd19*, *Il1b*, *Tnf*, *Duox2*, or *Nos2*, and carcinogenesis, including *Aurkb*, *Uhrf1*, *Cep55*, *Slc7a11*, and the 9 *Kif* genes, were spontaneously induced with the specific deletion of *Dhps* in IECS (Figure 4b), similar to our prior report.^26^ The most downregulated genes in *Dhps*^*∆epi*^ mice were *Kdm5d*, which encodes for a histone demethylase, and genes related to translation, such as *Eif2s3y* and *Rlp29*. Strikingly, among the 105 genes significantly upregulated in *C. rodentium*-infected *Dhps*^*∆epi*^ mice compared to their uninfected control, we found numerous markers of inflammation, such as *S100a8/a9*, *Nos2*, *Cxcl2*, *Ifng* (Figure 4c), even though the colon of naïve *Dhps*^*∆epi*^ mice already exhibited a pro-inflammatory/carcinogenic profile. Only 15 genes were downregulated with infection in *Dhps*^*∆epi*^ mice (Figure 4c). Lastly, we observed that genes that are markers of epithelial alteration (*Pbld1*, *Bach2*, *Col20a1/23a1/27a1/9a3*, *Wntf11*) are significantly upregulated in infected *Dhps*^*∆epi*^ mice versus infected *Dhps*^*fl/fl*^ animals (Figure 4d).

The global analysis of these transcriptomic changes was performed by IPA (Table S2). We evidenced that the “Canonical” pathways related to immune response, stress, injury, and disease were among the most upregulated in the colon of *C. rodentium*-infected *Dhps*^*∆epi*^ mice compared to the infected *Dhps*^*fl/fl*^ mice (Figure 4e). Further, in the category “Diseases & Functions”, the pathways belonging to “Organismal Injury and Abnormalities”, “Cancer”, and “Gastrointestinal Disease” were significantly induced by *Dhps* deletion (Figure S3).

#### Epithelial hypusination supports the detoxification of reactive aldehydes in infected mucosa

**Figure 5.**
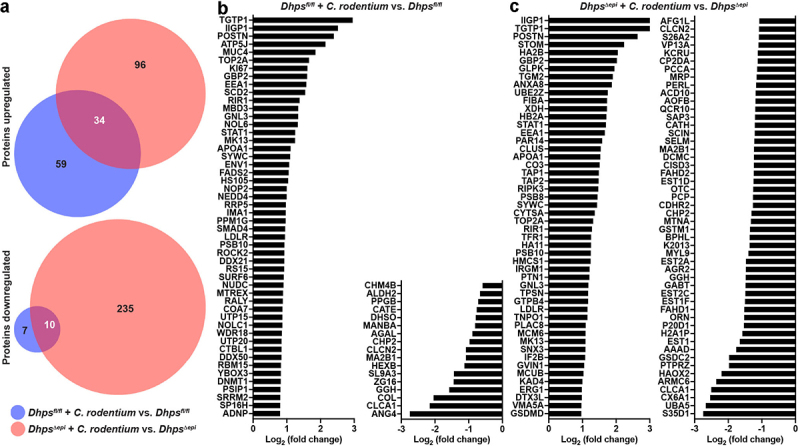
Effect of *C. rodentium* infection on the proteome of *Dhps*^*fl/fl*^ and *Dhps^Δ^*^*epi*^ mice. Label-free quantitative analysis was used to determine the differential expression of the proteins of isolated CECs from *Dhps*^*fl/fl*^ and *Dhps^Δ^*^*epi*^ mice infected or not with *C. rodentium* (*n* = 4 mice per group) for 14 days. The number of proteins significantly upregulated or downregulated in infected mice in both genotypes is depicted as a venn diagram (a). The proteome of CECs isolated from infected *Dhps*^*fl/fl*^ (b) and *Dhps*^*∆epi*^ (c) mice was compared to naïve animals. These figures depict the 50 proteins that are the most significantly upregulated or downregulated for each genotype. The complete list of proteins is given in Table S3.

To further determine the mechanism by which hypusination in IECs dampens *C. rodentium* colitis, we performed a proteomics analysis on isolated CECs from *Dhps*^*fl/fl*^ and *Dhps^Δ^*^*epi*^ mice infected or not with *C. rodentium* by label-free quantitative analysis, and a total of 3072 proteins were identified (Table S3). Overall, there was more proteins induced or downregulated significantly during the infection in *Dhps^Δ^*^*epi*^ versus *Dhps*^*fl/fl*^ mice, and only a minority of these proteins were common to both genotypes (Figure 5a). In CECs from *Dhps*^*fl/fl*^ mice infected with *C. rodentium*, 93 proteins were significantly upregulated compared to uninfected animals (Figure 5a and Table S3). The proteins induced included *i*) classical markers of the immune response, such as STAT1, APOA1P, POSTN, or SMAD4; but also *ii*) molecules involved in GTPase signaling (TGTP1, IIGP1, GBP2); *iii*) numerous proteins implicated in regulation of translation including IF6, EIF3H/3I/2A/3F/3F, XPO1, or DDX42; and *iv*) the proliferation marker KI67 (Figure 5b and Table S3). Note that among the 28 proteins undetected in CECs from control *Dhps*^*fl/fl*^ mice but present in infected mice, we found the pro-inflammatory markers I23O1, NOS2, and S10A8 (Table S3). Overall, proteins from the same families of signaling and pro-inflammatory markers were significantly induced in *C. rodentium*-infected *Dhps^Δ^*^*epi*^ mice (Figure 5c and Table S3). Only 17 proteins were significantly downregulated in CECs from *Dhps*^*fl/fl*^ mice during the infection, whereas 245, including 10 in common with *Dhps*^*fl/fl*^ mice, were less expressed in infected *Dhps^Δ^*^*epi*^ CECs (Figure 5a–c and Table S3).

**Figure 6.**
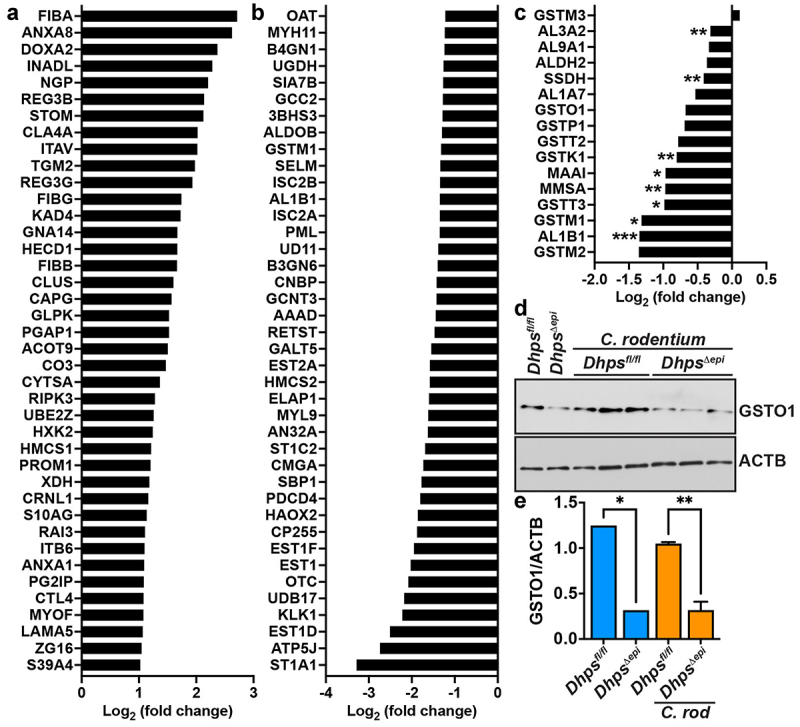
Effect of hypusination on the proteome of *C. rodentium*-infected CECs. *Dhps*^*fl/fl*^ and *Dhps^Δ^*^*epi*^ mice were infected or not with *C. rodentium* (*n* = 4 mice per group). Proteins were extracted from isolated CECs. Label-free quantitative analysis was performed and the 40 proteins that are the most significantly upregulated (A) or downregulated (B) in infected *Dhps^Δ^*^*epi*^ CECs compared to infected *Dhps*^*fl/fl*^ mice are shown. The complete list of proteins identified is provided in table S3. The proteins involved in aldehyde detoxification have been also identified in this analysis (C); *p < 0.05, **p < 0.01, ***p < 0.001. Proteins were also used to assess GSTO1 level by Western blot (D) followed by densitometric analyses (E); *p < 0.05, **p < 0.01 by ANOVA and Tukey test.

Interestingly, there were 144 proteins significantly more abundant in CECs from *C. rodentium*-infected *Dhps^Δ^*^*epi*^ mice compared to cells isolated from infected *Dhps*^*fl/fl*^ animals (Figure 6a and Table S3). These proteins were markers of inflamed mucosal tissues, such as the fibrinogens FIBA/B/G or the annexins ANXA8/A8, and the pro-inflammatory mediators DOXA2, APOA1, S10AG, or POSTN (Figure 6a and Table S3). These data indicate that *Dhps^Δ^*^*epi*^ mice exhibit exacerbated inflammation with *C. rodentium* infection when compared to floxed animals. We also discovered that 372 proteins were significantly less expressed in CECs from *Dhps^Δ^*^*epi*^ mice than in those from *Dhps*^*fl/fl*^ mice upon *C. rodentium* infection (Figure 6b and Table S3). These proteins mainly included metabolic enzymes, such as the sulfotransferases ST1A1 and ST1C2, and enzymes belonging to ornithine metabolism including the ornithine transcarbamylase (OTC), the ornithine aminotransferase (OAT), and the ornithine transporter 1 (ORNT1) (Figure 6b and Table S3). We previously reported that hypusination regulates the translation of numerous enzymes involved in detoxification of reactive aldehydes.^26^ Accordingly, we also observed that under conditions of *C. rodentium* infection, there were 8 proteins, including aldehyde dehydrogenases (ALDHs) and glutathione S-transferases (GSTs), significantly downregulated in *Dhps^Δ^*^*epi*^ mice versus *Dhps*^*fl/fl*^ mice (Figure 6c and Table S3); in addition, the expression of 7 other proteins with the same functionality were reduced in infected *Dhps^Δ^*^*epi*^ mice, but did not reach statistical significance (Figure 6c and Table S3). Lastly, thioredoxin (THIO) and the peroxiredoxins 6 (PRDX6), which are involved in peroxide detoxification and previously reported to be dampened in *Dhps^Δ^*^*epi*^ versus *Dhps*^*fl/fl*^ mice,^26^ were also significantly less expressed in infected *Dhps*^*∆epi*^ CECs (Table S3). We then confirmed by Western blotting (Figure 6d) and densitometry (Figure 6e) that the level of the protein GSTO1 was reduced in *Dhps*^*∆epi*^ mice, infected or not, compared to *Dhps*^*fl/fl*^ mice, thus validating the results obtained by proteomic analysis.

The differential proteomic dataset comparing *C. rodentium*-infected *Dhps*^*∆epi*^ to infected *Dhps*^*fl/fl*^ mice was filtered through the IPA pipeline to uncover the functional pathways regulated by hypusination in CECs during the infection. “Canonical” pathways related to immune response and bacterial pathogenicity were significantly induced by *Dhps* deletion (Figure S4A and Table S4). There was also a significant decrease of 37 pathways, including “Oxidative Phosphorylation”, “The Citric Acid (TCA) Cycle and Respiratory Electron Transport”, and “Glutathione-mediated Detoxification” (Figure S4A and Table S4). The “Diseases & Functions” pathways belonging to “Organismal Injury and Abnormalities”, “Cancer”, and “Inflammatory Response” were further induced in *Dhps*^*∆epi*^ mice compared to *Dhps*^*fl/fl*^ during *C. rodentium* infection (Figure S4B and Table S4).

Further, we found that the hypusination of EIF5A and the expression of GSTP1 were reduced in the human colonoid cell line DoD022, infected or not with EPEC, when pre-treated with the DHPS inhibitor GC7 (Figures S5A and S5B). GC7 had no effect on adherence and internalization of EPEC (Figure S5C).

#### Increased isoLG-lysyl adducts in infected *Dhps*^∆epi^ mice

**Figure 7.**
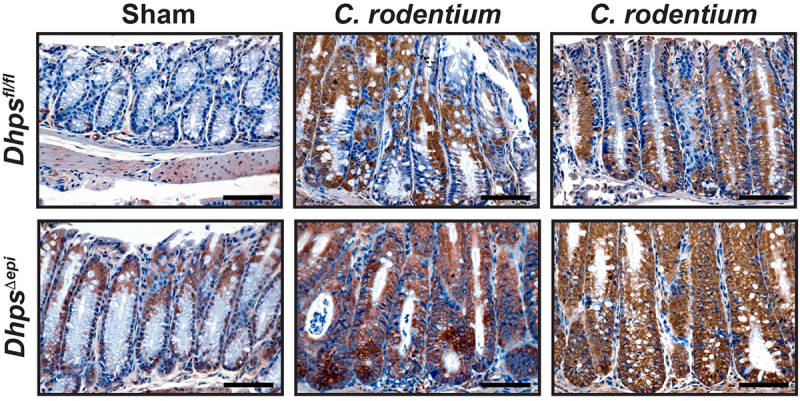
Levels of bifunctional electrophile adducts in infected mice. Colon from *Dhps*^*fl/fl*^ and *Dhps^Δ^*^*epi*^ mice infected with *C. rodentium* (*n* = 3 per genotype) or not (*n* = 5 per genotype) were immunostained with the D11 ab that detects isoLG-lysyl adducts. Representative images are shown. Scale bars represent 50 μm.

Enzymes that detoxify reactive aldehydes are less expressed in the colon of infected *Dhps*^*∆epi*^ mice (Figures 6c,e). Therefore, we thought that covalent adduction of these electrophiles, such as isoLGs, would be enhanced in CECs of mice specifically lacking *Dhps*. By IHC, we found increased isoLG adducts in CECs from naive *Dhps*^*∆epi*^ mice compared to *Dhps*^*fl/fl*^ animals (Figure 7), as we reported.^26^ Moreover, we observed an increase of isoLG-lysyl adducts in *Dhps*^*fl/fl*^ animals infected with *C. rodentium*, and a further enhancement in infected *Dhps*^*∆epi*^ mice (Figure 7).

## Discussion

The data presented herein demonstrates that hypusination in IECs protects from colitis induced by *C. rodentium*, an A/E pathogen that mimics human infection by EPEC. Because hypusination is enhanced in human and murine epithelial cells in response to EPEC and *C. rodentium*, respectively, we propose that this represents a critical post-translational strategy to counteract enteric infections.

We first observed that the protein DHPS and the level of hypusinated EIF5A are increased in the colon of mice infected with *C. rodentium*. Importantly, hypusination was also increased in the HCT 116 cell line and a human colonoid line in response to EPEC, underlining the human relevance of our findings. We found that increased hypusination level requires a functional T3SS but occurs independently of A/E lesions. Further, we observed that human IECs infected with a *nleA* mutant exhibited an increased level of EIF5A^Hyp^ compared to those infected with the parental strain or other mutants. Therefore, we propose that the inhibition of hypusination is a potential mechanism by which the virulence factor NleA supports the pathogenesis of A/E pathogens^37^; further investigations are warranted to determine the precise mechanism by which A/E pathogens regulate hypusination in IECs. Although we previously reported that this bacterium can stimulate DHPS induction in macrophages and increase the level of EIF5A^Hyp^ in CD68^+^ colonic cells,^24^ we contend that this induction also occurs in CECs since we found a marked reduction of both DHPS and EIF5A^Hyp^ in the mucosa of *Dhps*^*∆epi*^ mice. In contrast, we found reduced EIF5A^Hyp^ levels in the colon of patients with inflammatory bowel diseases and mice with dextran sodium sulfate (DSS) colitis.^26^ It will thus be interesting to understand the molecular etiology of this discrepancy between infectious colitis compared to noninfectious etiologies. Nonetheless, supporting the concept that hypusination is an essential feature of cells exposed to an infection, it has been shown that microvascular endothelial cells infected with Kaposi’s sarcoma-associated herpesvirus^38^ or group 3 innate lymphocytes treated with *Clostridium difficile* TcdB toxin^39^ exhibit an increase of EIF5A^Hyp^ level.

We showed that mice with specific deletion of *Dhps* in IECs exhibit increased susceptibility to *C. rodentium*. First, we found increased colonization in *Dhps*^*∆epi*^ mice. A similar phenotype was observed in mice with specific deletion of *Dhps* in myeloid cells.^24^ We can therefore suggest that hypusination in both epithelial and myeloid cells leads to the control of the infection. However, as expected, the proteome of infected macrophages^24^ differs from that of infected CECs, shown herein, and no major antibacterial effectors were found to be regulated by hypusination in CECs from infected mice. This suggests that the control of the infection by epithelial hypusination occurs through the induction of an inflammatory state, which results in the recruitment of professional immune cells, rather than by a direct antibacterial effect of the epithelium. But overall, it is interesting to observe that hypusination in the colon mucosa controls the burden of enteropathogenic bacteria. Secondly, we found increased inflammation and histological damage in infected *Dhps*^*∆epi*^ mice. Again, the same phenotype was observed in *Dhps*^*∆mye*^ mice,^24^ demonstrating that hypusination in different cells in the colon is critical to reduce colitis in response to pathogens.

We previously described that *Dhps*^*∆epi*^ mice develop spontaneous colon inflammation and are highly susceptible to DSS colitis, notably due to the translation of enzymes involved in aldehyde detoxification and increased levels of electrophile adducts in CECs,^26^ a hallmark of gastrointestinal tissue damage.^26,40–42^ Interestingly, we confirmed in the present paper that DHPS in IECs supports the translation of proteins involved in detoxification of peroxides (THIO, PRDX6) and reactive aldehydes (ALDHs, GSTs) during *C. rodentium* infection. Consequently, *Dhps*^*∆epi*^ mice with reduced hypusination exhibited a marked increase of electrophile adducts in the colonic mucosa, which is consistent with exacerbated colitis. However, the protective role of IEC DHPS in this model is certainly multifactorial and we are pursuing efforts to evidence other molecular and cellular pathways regulated by hypusination that support intestinal homeostasis. Altogether, these data support the contention that the DHPS/hypusine pathway in epithelial cells is essential to prevent intestinal inflammation associated with different etiologies.

The composition of the gut microbiota can affect *C. rodentium* colonization and pathogenicity.^43,44^ However, we previously reported that *Dhps* deletion does not affect diversity and richness of the microbiota and does not significantly alter the microbiome at the phylum and genus levels.^26^ It is therefore unlikely that the worsening of the inflammation in *Dhps*^*∆epi*^ mice results from an effect on the intestinal microbiota.

The data presented herein suggest that the hypusination pathway is essential to protect humans against EPEC-induced colitis. But this topic deserves further clinical investigation, including determining *i*) the level of hypusination in the colon of EPEC-infected patients, and *ii*) whether increased hypusination is a fast and transitory response to the infection to limit colonization and/or a chronic mechanism developed by the host to protect the colon, and *iii*) whether patients with reduced levels of the DHPS/EIF5A^Hyp^ metabolic pathway are more prone to develop severe disease related to EPEC infection. Consequently, restoration of hypusination could represent a novel therapeutic approach. The polyamine Spd exhibits numerous beneficial properties in the cardiovascular system,^45^ reduces neurodegeneration,^46^ and exhibits anti-inflammatory and anti-tumoral properties in the gastrointestinal tract.^47^ Moreover, we found that deletion of the gene *Smox*, encoding for spermine oxidase, the enzyme that generates Spd from spermine, exacerbates colitis and colitis-associated carcinogenesis^13,47^ and that treatment with Spd restores hypusination and dampens colon inflammation and carcinogenesis.^47^ However, in contrast, deletion of *Smox* protects mice from *C. rodentium*-induced colitis, suggesting that Spd supplementation could be deleterious in patients with A/E pathogen infection. In this context, our current data further highlight the complexity and the heterogeneity of the role of the polyamine pathway in diseases but demonstrate that hypusine protects from infection-induced colitis.

## Supplementary Material

Supplemental Material

## Data Availability

The authors confirm that the data supporting the findings of this study are available within the article and its supplementary materials. RNA-Seq data can be accessed on the GEO repository using the accession number GSE262899. The mass spectrometry proteomics data have been deposited to the ProteomeXchange Consortium via the PRIDE partner repository with the dataset identifier PXD051536.
