# Supplementary material for: Hypusination in intestinal epithelial cells protects mice from infectious colitis
Source: Gut Microbes. 2024 Dec 14;16(1):2438828. doi: 10.1080/19490976.2024.2438828 (PMC11649231; doi:10.1080/19490976.2024.2438828)
Supplement: Supplemental Material [file KGMI_A_2438828_SM2431.zip › SuppFigures_3.docx]

**Females**

✱✱✱✱


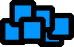

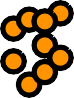


✱✱✱

✱✱

✱✱✱

**Males**

**0.06**

ns

**Weight/length (g/cm)**

**0.04**

**0.02**

**0.00**

✱✱

**0.08**

ns


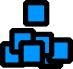


✱

✱✱

**Weight/length (g/cm)**

**0.06**

**0.04**

**0.02**

**0.00**

✱✱

✱✱✱✱

ns

ns

ns


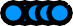

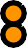

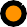


✱✱

✱

✱


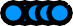

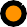


✱

✱✱✱

**25 25**

**20 20**

**Histologic injury score**

**Histologic injury score**

**15 15**

**10 10**

**5 5**

**0 0**

## C. rod C. rod

**Figure S1.** *C. rodentium* colitis in males and females. *Dhps^fl/fl^* and *Dhps^∆epi^* mice were infected with *C. rodentium* and sacrificed after 14 days. The colon weight/length ratio and histologic injury score were determined and plotted for each gender. **P* < 0.05, ***P* < 0.01, ****P* < 0.001, *****P* < 0.0001 by one-way ANOVA and Newman-Keuls test.

**A *Dhpsfl/fl Dhps***Δ***epi***


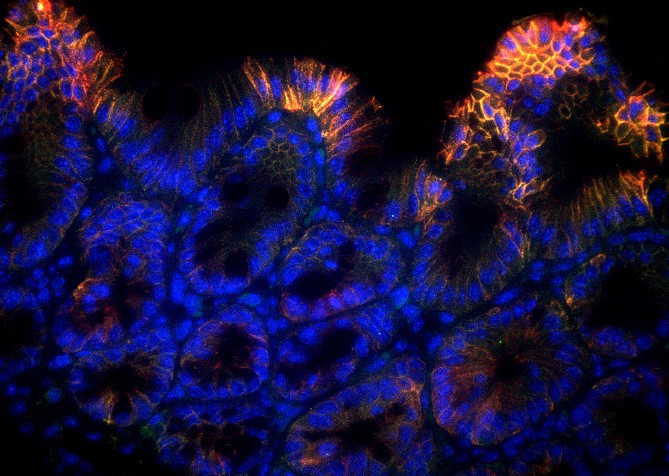

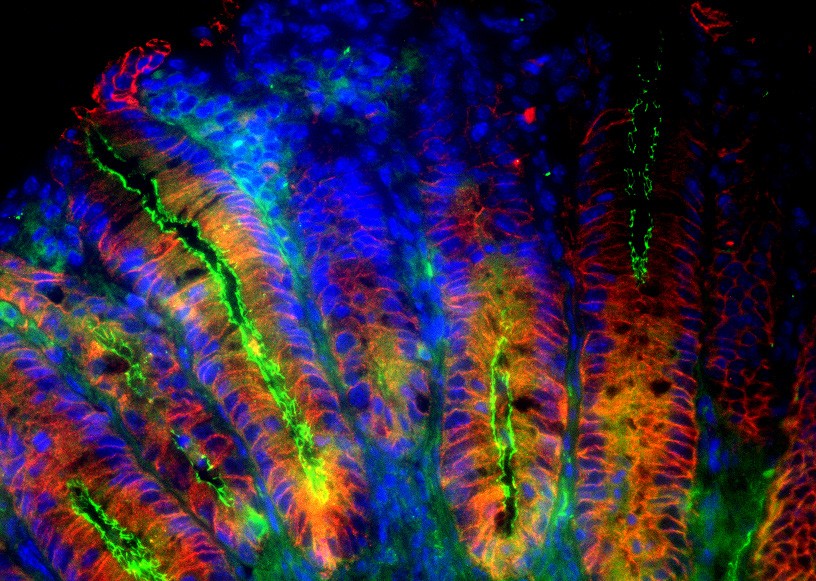

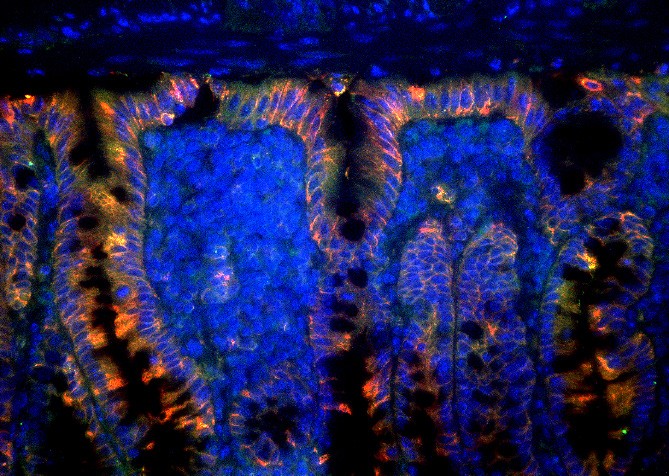

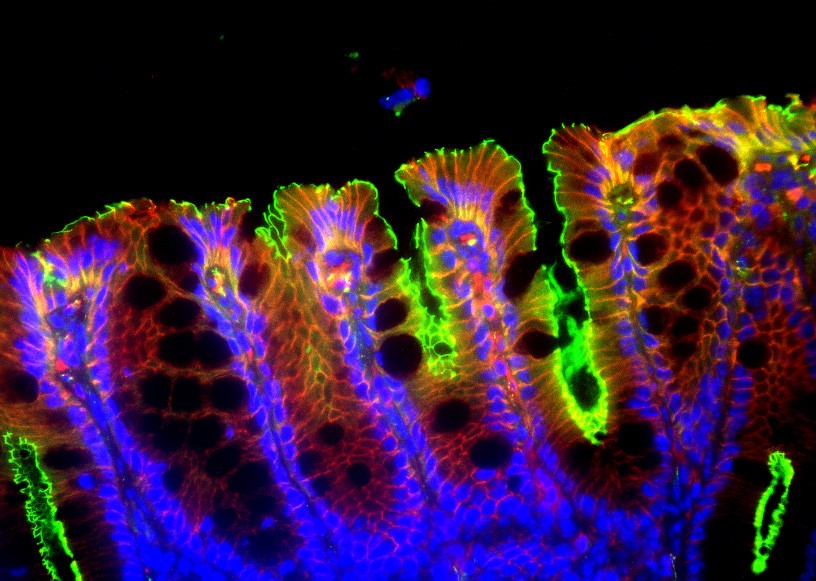


**Sham**

**B**

**Number of cases (%)**

***C. rodentium***

**100**

**8**

**6**

**4**

**5**

**75**

**50**

**25**

**0**

***P* = 0.6802**

**C**

# C. rod. 6


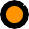

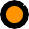

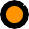

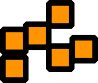


***C. rodentium***

**Log (CFU/g tissue)**

**no *C. rod.***

**4**

**2**

**0**

# C. rod C. rod

**Figure S2.** Effect of *Dhps* deletion in IECs on the integrity of the intestinal epithelial barrier. *Dhps^fl/fl^* and *Dhps^∆epi^* mice were infected or not with *C. rodentium* for 13 days. (A) ZO-1 (green) and E-cadherin (red) were immunodetected in the colon; nuclei are in blue. The images are representatives of 3 uninfected and 5 infected mice per genotype. Scale bars, 50 μm. (B) The number of animals with live *C. rodentium* in the spleen was assessed by plating serial dilutions of ground spleens; *P* was calculated by Fisher's exact test. (C) The same method was used to determine the absolute quantification of bacteria in the spleen of animals harboring splenic colonization.


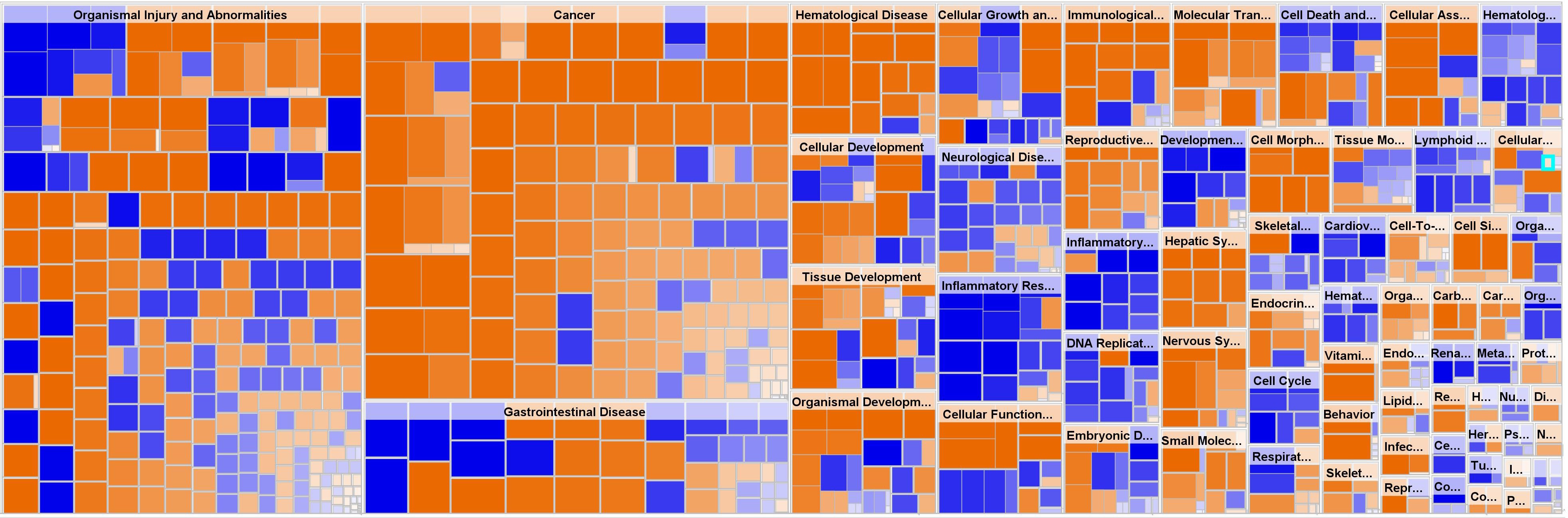
***Dhps***Δ***epi* + *C. rod* vs. *Dhpsfl/fl* + *C. rod***

**-2 +2**


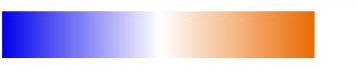
**z-score**

**Figure S3.** Pathways related to Disease & Functions determined by IPA performed on RNA-Seq. The complete list of pathways is provided in Table S2.

**A**


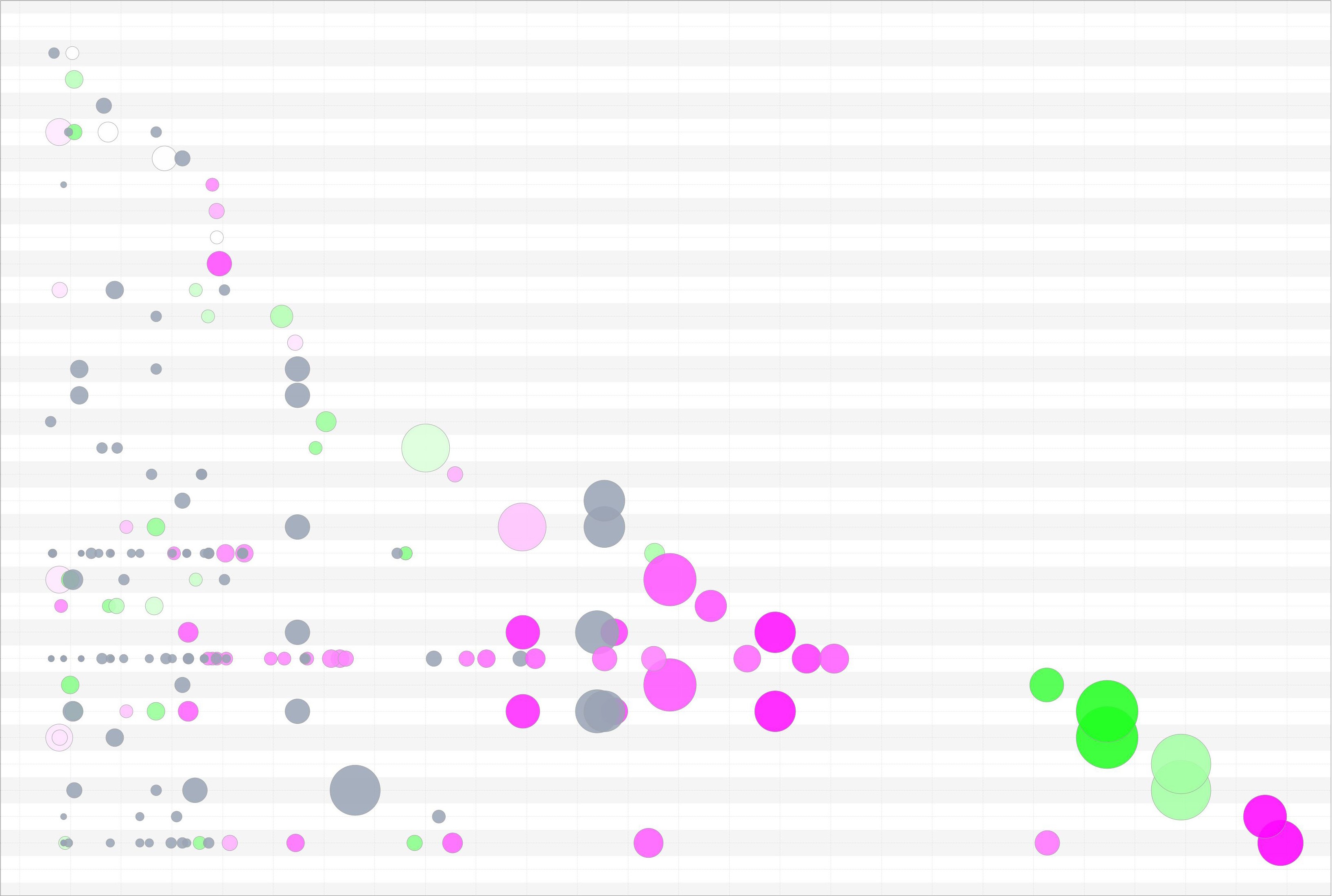

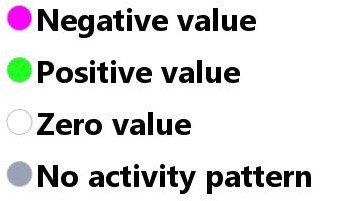

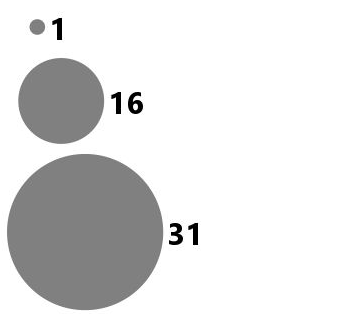


**z < 0**

**z > 0**

**z = 0**

**No activity pattern**

**Number of genes that overlap the pathway 1**

**16**

**31**

**Developmental Biology Cytokine signaling**

**Neurotransmitters and other nervous sytem signaling Cellular growth, proliferation and development Organismal growth and development Glutathione-mediated detoxification**

**Protein localization Cellular responses to stimuli Vesicle-mediated transport Cardiovascular signaling**

**Hemostasis Muscle contraction Cell cycle regulation**

**Apoptosis**

**Extracellular matrix organization**

**Immune system**

**Signal transduction Pathogen-influenced signaling Nuclear receptor signaling**

**Biosynthesis Cellular stress and injury Metabolism of proteins Xenobioticmetabolism Degradation/utilization/assimilation Cellular Immune response**

**Ingenuity toxicity Disease-specific pathway Transcriptional regulation**

**Intracellular and second messenger signaling Generation of precursormetabolites and energy**

**Metabolism**

**1.0 2.0 3.0 4.0 5.0**

**B**


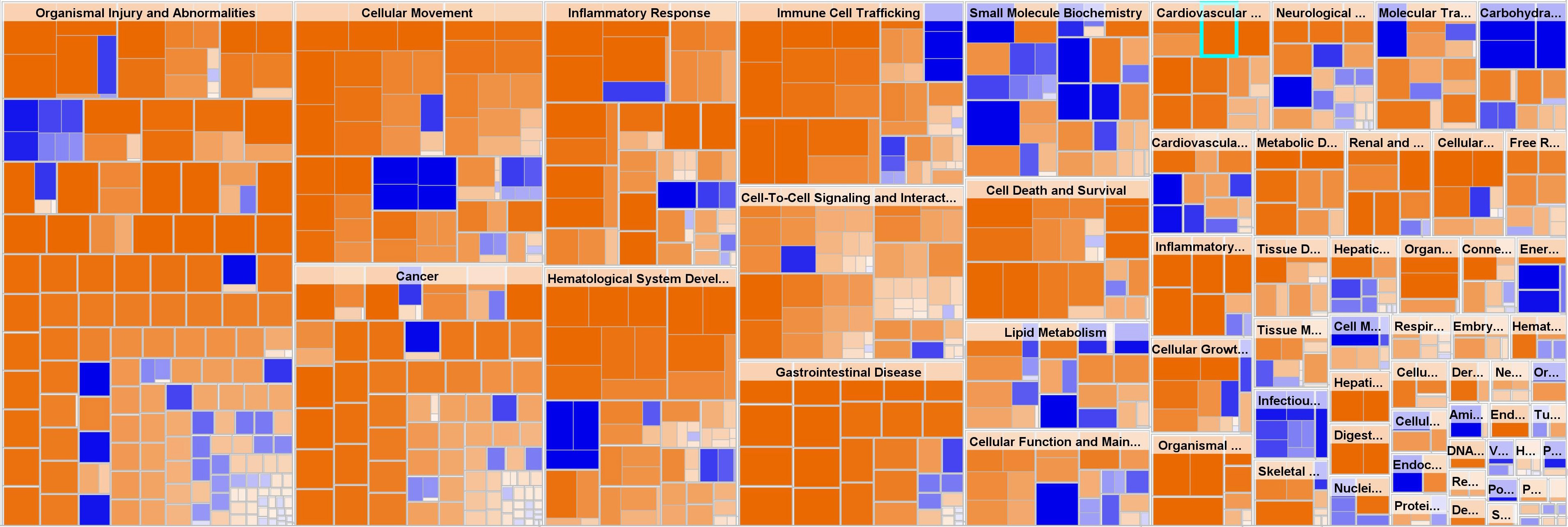

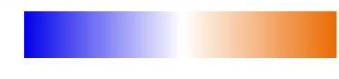


**z-score**

**-2 +2**

**6.0 7.0 8.0 9.0**

**-Log10 (*P*)**

**10.0**

**11.0**

**12.0**

**13.0**

**Figure S4.** Functionality of the proteome differentially affected in *Dhps^fl/fl^* and *Dhps^∆epi^* mice during *C. rodentium* infection. IPA using the differential proteomic dataset comparing *C. rodentium*-infected *Dhps^∆epi^* to infected *Dhps^fl/fl^* mice was used to determine the “Canonical” (A) and the “Disease & Functions” pathways. See Table S4 for the complete list of pathways.

### A B _✱_

#### EPEC – – + +

**EIF5AHyp/EIF5A/ACTB (X105)**

**GC7 – + –**


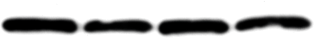

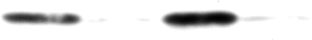

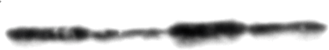

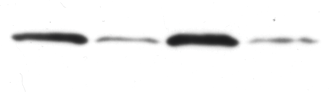


**+**

**15**


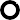

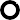

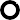

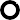

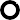

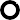

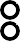

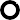

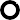

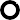


**EIF5AHyp**

**10**

#### EIF5A

**5**

#### GSTP1

**0**

✱✱

**1.0**


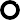


**GSTP1/ACTB**

**0.8**

**0.6**

**0.4**

**0.2**

**0.0**

✱✱✱✱

**EPEC – – + + EPEC – – + +**

### C

**Number of EPEC/cell**

**0.15**

**0.10**

**0.05**

**0.00**

#### ACTB

**GC7 – + – +**

**30**


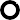

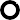

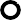

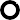

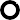

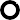

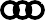

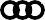


**1 hr**


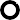

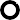


**6 hr**

**20**

**10**

**6**

**3**

**0**

#### GC7 – + – +

**GC7 – +**

**– +**

**– +**  **– +**

**Gentamicin Gentamicin**

**Figure S5.** Regulation of GSTP1 by hypusination in human colonoids. The level of EIF5A^Hyp^, EIF5A, and GSTP1 was assessed by Western blot in the in the normal human colonoid line DoD022 pretreated with GC7 for 2 h and infected or not with EPEC for 6 h (A). The densitometric analysis is shown in (B); **P* < 0.05, ***P* < 0.01, and

****P* < 0.001 by ANOVA and Tukey test. (C) Attachment and intracellular survival of EPEC (+ Gentamicin) in CECs determined by culture of serial dilutions; each dot represents an independent experiment.
